# Study on electromagnetic characteristics of cylindrical hole defect in variable parameter traction motor shaft based on eddy current effect

**DOI:** 10.1371/journal.pone.0342673

**Published:** 2026-02-17

**Authors:** Mengmeng Song, Tingting Zhu, Shungen Xiao, Mengwei Li, Gentao Lai, Zhenhao Cai, Qing Li, Zhigang Xu

**Affiliations:** 1 Ningde Normal University, School of Information Engineering, Ningde, People’s Republic of China; 2 Fujian Agriculture and Forestry University, School of Engineering, Fuzhou, People’s Republic of China; 3 Fuzhou University, School of Mechanical Engineering and Automation, Fuzhou, People’s Republic of China; 4 Ningde Sikeqi Intelligent Equipment Co., Ltd., Ningde, People’s Republic of China; 5 Longyan University, School of Resource Engineering, Longyan, People’s Republic of China; Zhejiang University, CHINA

## Abstract

Detecting common cylindrical hole defects (corrosion defects) in locomotive traction motor shafts is essential to ensure equipment reliability and safety. This paper conducts an in-depth study of rotating shafts with a rich magnetic field surrounding them, with the goal of identifying cylindrical hole defects and diagnosing corrosion defects. During eddy current testing, to investigate the influence of variable-parameter (bottom diameter and depth) cylindrical hole defects on the electromagnetic properties of traction motor shafts, an equivalent model of a variable-parameter defect detection system for cylindrical hole defects (corrosion defects) was established using COMSOL software, based on eddy current testing theory, and simulation analysis was performed. By studying the horizontal and vertical magnetic induction intensities(HMII, VMII) and their respective phases(PHMII, PVMII), the magnetic field distribution around the cylindrical hole defect under variable parameters was analyzed. The results show that HMII and its phase have a significant geometric correspondence, allowing for quantitative assessment of the defect bottom diameter through characteristic peak spacing or phase lag width. Simultaneously, the amplitude variations of HMII and VMII can qualitatively determine the presence of defects and evaluate their relative depth (limited by the saturation threshold). Furthermore, PVMII is insensitive to the defect’s geometric parameters.

## 1. Introduction

In many engineering construction projects in society, mechanical devices will bear certain loads for a long time, and the key components will be in harsh working environments such as temperature, humidity, high pressure, and high dust all year round. The mechanical properties will degrade exponentially with the service life, resulting in equipment failure and failure to operate normally. Usually, cracks and corrosion defects in the equipment are the main causes of equipment failure. The role of non-destructive testing technology is very prominent. It can detect defects in the equipment in a timely manner and ensure the normal operation of the equipment. At present, this technology includes magnetic particle testing (MT), ultrasonic testing (UT), eddy current testing (ECT), radiographic testing (RT) and penetrant testing (PT). Non-destructive testing uses electrical, magnetic, acoustic, optical, and radiographic means to detect the physical and chemical properties and organizational morphology of the test piece. With the development of modern industry, non-destructive testing has become an important testing method for product quality control and quality assurance [1]. One of the most commonly used technologies is ECT, which is based on the principle of electromagnetic induction and is often used to detect the internal and surface structural conditions of the tested parts. It evaluates the changes in electromagnetic field parameters and has the advantages of fast detection speed, low detection surface requirements, no damage to the tested parts, no need for contact and coupling agents, convenient operation, and relatively friendly to the human body. It is indispensable in the field of parts detection, usually detecting metal parts to ensure product quality and structural integrity [2].

Eddy current detection technology was first put into practical use in 1879. Eddy current detection technology was used by Karnz and Farraw to detect pipe wall thickness and flaws, which enabled eddy current detection technology to be applied in new fields [3]. In the middle of the last century, German scientist Foster pioneered the impedance analysis method. This new theory strongly supported the application of eddy current detection [4]. In the 21st century, Machado et al. proposed an eddy current detection (ECT) detection system for surface and subsurface welding defects integrated with a robot arm. Through experimental verification, it can detect porosity defects and hidden depth defects [5]. In order to overcome or reduce the influence of the skin effect in eddy current detection, Sergeeva-Chollet et al. combined the magnetoresistive sensor array with the traditional eddy current probe with the help of finite element simulation [6]. Ye C et al. used TMR sensors to measure the weak low-frequency magnetic field signals caused by deep defects, and quantitatively characterized underground defects with spatial and frequency domain characteristics [7]. Rajamäki J et al. concluded through research that penetration depth is important in eddy current testing, and that depth testing is more susceptible to changes in electromagnetic properties [8]. Binghua C et al. established an analytical model of the eddy current field under sinusoidal excitation of a planar spiral coil, and verified the model through finite element and experimental results. The results showed that the frequency and dielectric constant of the sample would affect the eddy current signal [9]. Mizukami et al. studied an eddy current method for evaluating the corrugation size of carbon fiber reinforced plastics. The results showed that the corrugation shape was manifested as a deformation of the magnetic field distribution. Finite element analysis was performed to study the relationship between the corrugation size and the magnetic field deformation size. The corrugation length can be accurately estimated from the length of the magnetic field deformation [10].Wen Simin et al. used an array eddy current non-destructive testing method based on the array structure of spin sensing and magnetic shielding structure, which greatly improved the detection efficiency while ensuring the detection accuracy [11]. Xiong Longhui et al. applied eddy current detection technology to the identification of scratches on high-speed railway steel rails, and analyzed the influence of different rail surface conditions on the scratch discrimination of eddy current detection signals [12]. Chen Hongfu et al. conducted surface defect detection experiments on the bearing roller, the most important component of rolling bearings, designed and built a bearing roller eddy current detection system, and compared two different characterization methods, voltage characterization and mutual inductance characterization, and concluded that the mutual inductance characterization method is better [13]. Xiao Fei et al. applied eddy current detection technology to the lead sealing condition detection of high-voltage cables [14]. Li Linfeng et al. used the finite element method to analyze the magnetic field distribution under parametric simulation of rail damage, and realized the forward analytical solution and simulation solution of rail damage by eddy current non-destructive testing technology, and studied the change of magnetic induction intensity and the identification of crack depth and width [15]. Zeng Huiyao et al. used the finite element model to simulate and calculate the change of the induced voltage signal output by the vertical eddy current detection probe when there are delamination defects of different thicknesses. The simulation results show that the analysis of the change of induced voltage can realize the quantitative evaluation of the thickness and range of delamination defects of unidirectional carbon fiber composite materials [16]. Ren Shuting et al. proposed a magnetic field gradient pulse eddy current detection method, which is effectively applied to the detection and evaluation of structural corrosion defects and can quantitatively evaluate the depth, morphology, opening size, etc. of subsurface corrosion defects of metal structures [17]. Wang Xin et al. used the pulse eddy current detection method to detect small dot-type pitting corrosion defects on the back of ferromagnetic flat components [18]. Jiang Feng proposed a method for quantitatively evaluating cylindrical corrosion defects using the lateral impact nodes and longitudinal depression depth in the butterfly diagram [19]. A crack conductivity distribution model was proposed by Li W et al., which used ECT technology to quantitatively evaluate the corrosion defect profile [20]. Li Zhonghu et al. created a pipeline inner wall corrosion defect model for cylindrical corrosion defects. According to the results of electromagnetic field simulation research, as the defect depth increases, the impedance amplitude has a linear relationship with the corrosion defect [21]. Furthermore, the acoustic wave detection model based on the energy variational principle proposed in reference [22] creatively treats defects as energy sources. This energy-based dynamic description of defect perturbations is highly consistent with the physical mechanism of eddy current fields being distorted by defect impedance, providing an important interdisciplinary new perspective for understanding the nature of defects.

However, due to the continuous innovation and breakthrough of modern detection technology, for example, Ren et al. utilized pulsed eddy current, and Li et al. based their work on impedance amplitude analysis, laying the foundation for quantitative defect evaluation. However, facing increasingly stringent and precise inspection requirements, modern equipment has established new benchmarks for defect detection, thus highlighting the shortcomings of traditional eddy current testing technology, and modern equipment has a new benchmark for defect detection, so the shortcomings of traditional eddy current detection technology continue to emerge. The reasons include that the application of non-destructive testing technology is becoming more and more popular. Existing studies are mostly limited to scalar changes in coil impedance or induced voltage, lacking a systematic analysis of the spatial distribution patterns of the magnetic induction intensity vector components (HMII and VMII) and their phases in three-dimensional space. Furthermore, most studies rely on amplitude regression for quantitative analysis, with few reports utilizing phase geometric features (such as the spatial width of the phase lag region) for direct size inversion. In addition, the traction motor shaft, as a rotating component with a specific curvature, exhibits different surface electromagnetic field distributions compared to flat or pipe structures. Therefore, traditional ECT technology is limited to detecting and diagnosing the existence of defects. It has poor adaptability to complex working conditions and suffers from a bottleneck in sensitivity. It can no longer meet the needs of today’s society and cannot achieve accurate diagnosis. In addition, tiny defects are difficult to detect [23]. For this reason, accurate diagnosis of micro defects has become a frontier research hotspot in the field of non-destructive testing. It is urgent to study and analyze the magnetic field distribution around tiny defects, so as to achieve the purpose of accurate detection and explore the relationship between the MF parameters around the defects and the cylindrical hole defects. In short, in-depth research on the magnetic field distribution around micro defects and establishing a quantitative relationship between magnetic field parameters and cylindrical hole defects are the only way to break through the bottleneck of traditional ECT.

In this regard, this paper selects the traction motor shaft commonly used in the field of transportation as the research object. In many common road construction or urban planning and construction projects, the traction motor is responsible for the traction work of the locomotive or EMU. When the locomotive is running, the rotating shaft is not only subjected to harsh working conditions involving high humidity, dust, and strong vibrations, but also experiences bending and torsional moments simultaneously, leading to the generation of bending and torsional stresses. Unlike static pipe or plate structures, even small corrosion defects on the rotating shaft, if not detected in time, can easily propagate rapidly into fatigue failure under high-speed rotation and alternating loads, seriously jeopardizing operational safety. As a key component for the motor to transmit torque, the shaft is an important load-bearing tool. Therefore, the health of the shaft cannot be ignored and directly affects the safety and life of the train [24–25]. The surface corrosion defects of the shaft need to be diagnosed and evaluated in time to ensure its safety and reliability. It is important to note that although the traction motor shaft in actual operation is subjected to complex environmental influences such as temperature changes, high-speed rotation, and strong vibrations, in order to isolate environmental noise interference and accurately investigate the fundamental influence of the defect geometry itself on the electromagnetic field distribution, this paper focuses on controlled steady-state conditions. This aims to establish a pure defect-magnetic field mapping baseline, laying a theoretical foundation for subsequent dynamic monitoring studies that incorporate complex operating condition variables. Based on the eddy current detection theory, the equivalent model of the variable parameter defect detection system of the cylindrical hole defect (corrosion defect) was established using COMSOL software, and simulation analysis was performed. The relationship between MF and the defect was analyzed by observing the horizontal magnetic induction intensity (HMII), vertical magnetic induction intensity (VMII) and their respective phases (PHMII and PVMII) of the MF in the space near the defect.

## 2. Eddy current testing theory

### 2.1. Eddy current testing principle

**Fig 1:**
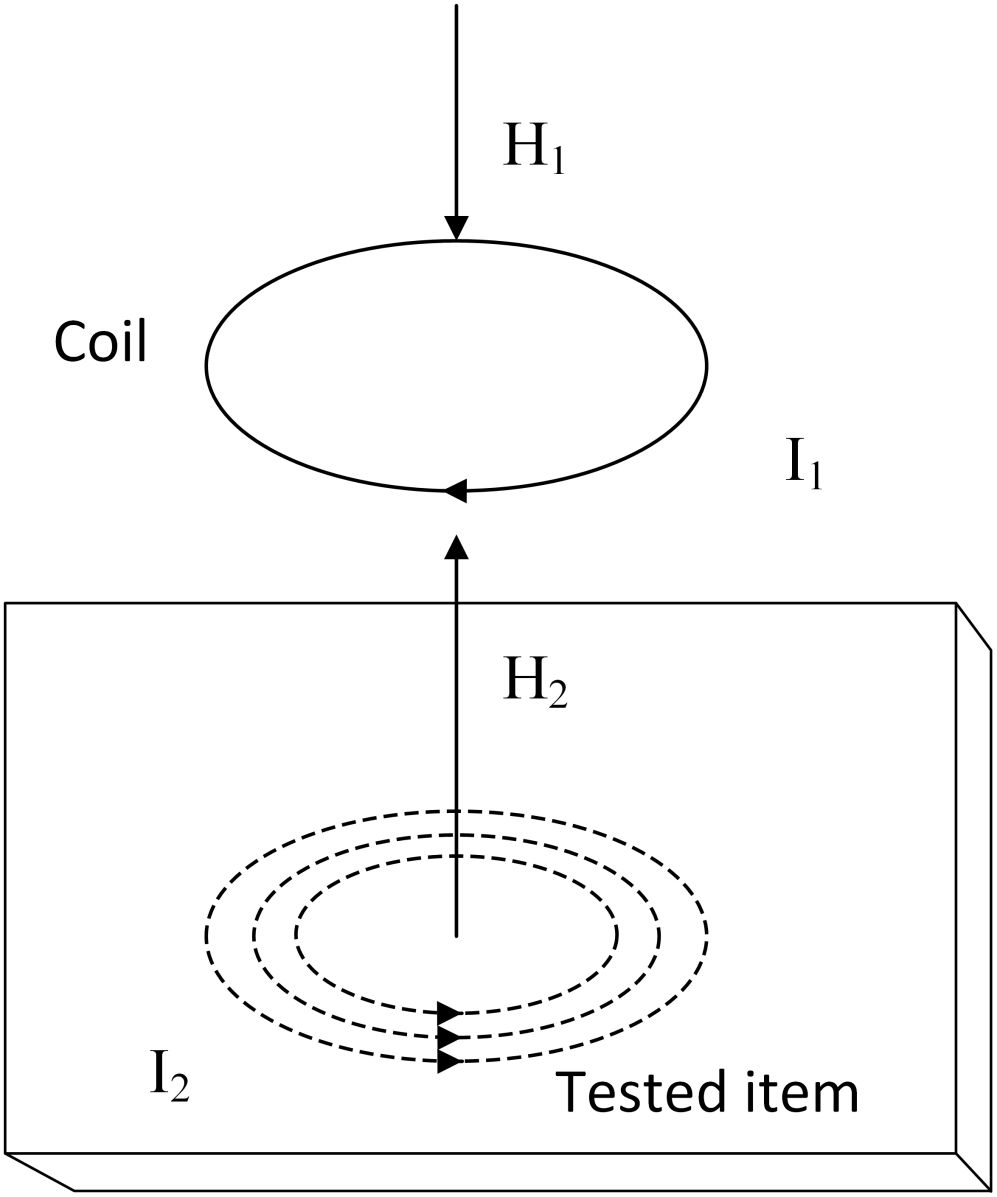
Schematic diagram of eddy current detection.

An important application of the eddy current effect is eddy current detection. The principle of eddy current detection is shown in Fig 1. ECT technology is based on the principle of electromagnetic induction. When a coil with alternating current is placed close to the metal surface, an alternating magnetic field (MF) will be generated around it. The coil will induce a circle of current like a “vortex” on the metal surface: this is eddy current [26]. This will generate a magnetic field that causes the impedance of the probe coil to change, and this magnetic field will affect the surface state of the specimen. When the metal is intact, the distribution and size of the eddy current are fixed. Once cracks, corrosion or material changes (i.e., “defects”) occur, the direction and density of the eddy current will change, which in turn affects the impedance in the coil. By measuring the coil impedance, the defects of the specimen can be detected, and the performance of the specimen can be evaluated. This influence law can detect the surface state of the specimen and judge its defect situation.

Maxwell’s equations consist of four equations: Ampere’s law of total current, Faraday’s law of electromagnetic induction, Gauss’s law of magnetic flux, and the law of magnetic flux continuity. The differential form of Maxwell’s equations can be written as [27]:


$$\nabla \times H=J+\frac{\partial D}{\partial t}$$ (2-1)

$$\nabla \times E=-\frac{\partial B}{\partial t}$$ (2-2)

∇·B=0 (2-3)

∇·D=ρ (2-4)

In equations (2–1) to (2–4), H is the magnetic field intensity (A/m); E is the electric field intensity (C/m²); J is the current density (A/m²); D is the electric field intensity vector (C/m²); B is the magnetic field intensity vector (T); ρ is the current density (C/m^3^); ∇· is the divergence operator; ∇× and is the curl operator. Because the motion time of free charges is very short, the current density of metallic conductive materials is set to 0.

Before solving the time-harmonic electromagnetic field partial differential equations, vector magnetic potential A and scalar magnetic potential Φ are usually introduced to simplify the iterative calculation:


B=∇×A (2-5)

E=−∇×Φ (2-6)

Before deriving the electromagnetic relationship in the eddy current region, the medium equation should be solved jointly:


$$\left\{ \begin{array}{c} B=\mu H;\\ D=\varepsilon E;\\ J=\sigma E \end{array}\right.$$ (2-7)

In the formula: *ε* is the dielectric constant (F/m); *μ* is the magnetic permeability (H/m); *σ* is the electrical conductivity (Ω^-1^ ·m^-1^).

Obtaining the vector relationship of the magnetic field in the eddy current detection system, all vectors A in the equation always have:


∇·(∇×A)=0 (2-8)

Formula (2–8) with formula (2–3) in Maxwell’s equations, we can get:


B=∇×A (2-9)

Substituting formula (2–9) into the formula ∇×E=−jBω, we can get:


∇×(E+jωA)=0 (2-10)

In the electromagnetic vector relationship, any scalar function contained in the electromagnetic field φ must have:


∇×(∇φ)=0 (2-11)

Looking at equations (2–10) and (2–11), we can get:


E+jωA+∇φ=0 (2-12)

According to the vector relationship $\nabla \times \nabla \times P=\nabla (\nabla \cdot P)-\nabla^{\text{2}}P$,


$$\nabla^{3}A+k^{2}A=-\mu J_{s}+\nabla \left(\nabla \cdot A-\frac{k^{2}}{j\omega }\varphi \right)$$ (2-13)

Where, $k^{2}=-\mu j\omega \left(\sigma +j\omega \varepsilon \right)$.

With the help of formula (2–7), formula (2–12) and formula (2–4), we can get:


$$\nabla^{2}\varphi +j\omega \nabla \cdot A=0$$ (2-14)

In order to make Equation (2–8) easier to solve, the electromagnetic potential function must (A,φ) satisfy the Lorentz specification:


$$\nabla \cdot A-\frac{k^{2}}{j\omega }\varphi =\text{0}$$ (2-15)

Using this specification, equations (2–13) and (2–14) can be simplified to:


$$\nabla^{3}A+k^{2}A+\mu J_{s}=0$$ (2-16)

$$\nabla^{2}\varphi +k^{2}\varphi =0$$ (2-17)

A is obtained, the expression of the scalar potential is obtained by using the Lorentz canonical form Φ:


$$\varphi =\frac{j\omega }{k^{2}}\nabla \cdot A$$ (2-18)

Substituting into formula (2–12), we can get that the electric field intensity can be expressed by vector magnetic potential:


$$E=-j\omega A-\frac{j\omega }{k^{2}}\nabla \left(\nabla \cdot A\right)$$ (2-19)

Equations (2–9) and (2–19) show that as long as A can be determined, the various components of the electromagnetic field can be determined.

### 2.2 Impedance analysis

**Fig 2:**
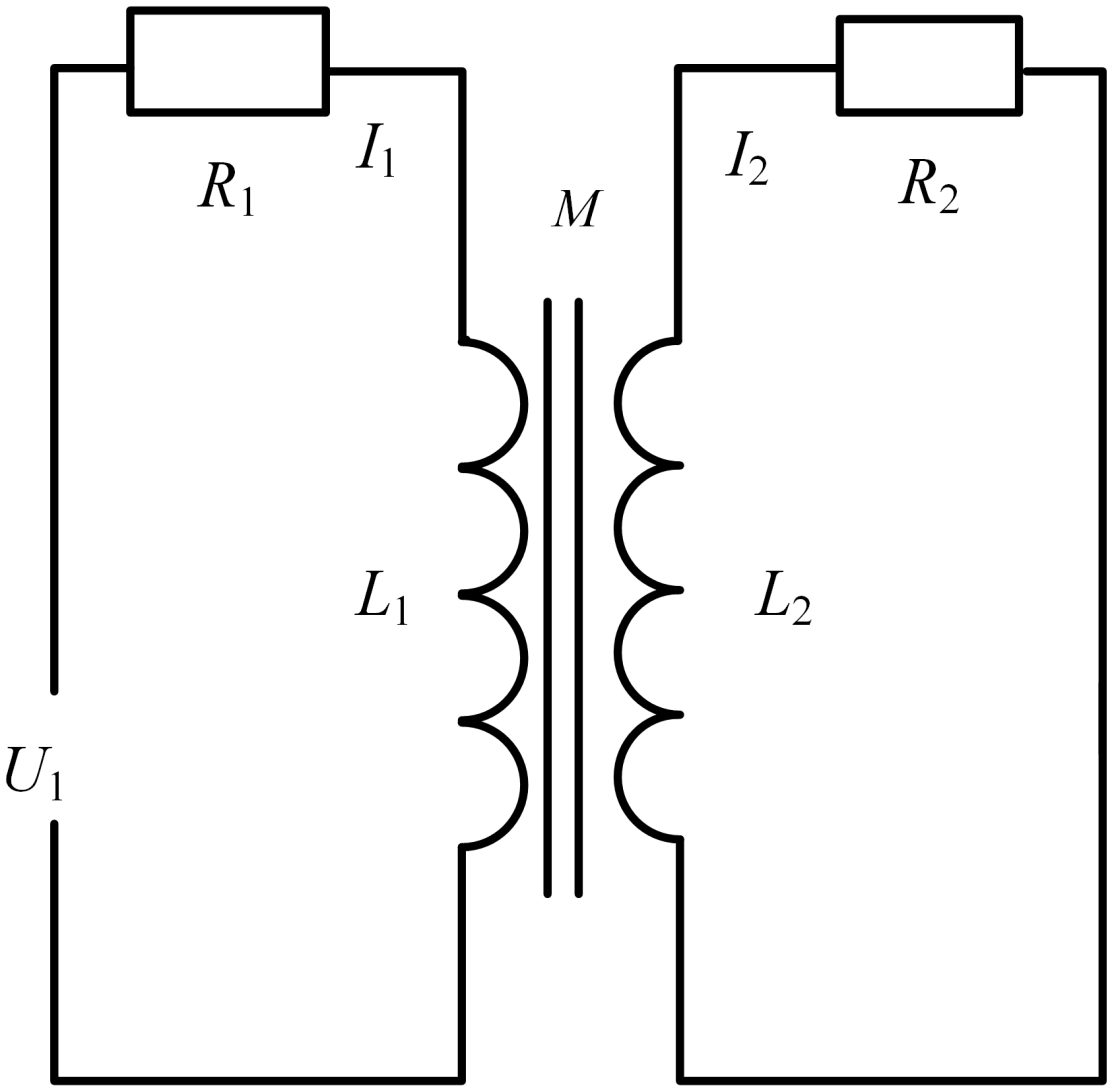
Equivalent circuit diagram.

The impedance analysis method was introduced, which is widely used in current eddy current detection projects. The equivalent circuit diagram formed by coil coupling is shown in Fig 2.

In the Fig 2: R1 and R2 are the resistances of the probe coil and the DUT, respectively; the pads detect the inductance L1 and L2 of the probe coil and the DUT; M is the mutual inductance between the probe coil and the DUT; U1 is the excitation voltage across the probe coil.

According to Kirchhoff’s voltage law, the voltage equations of the original circuit and the secondary circuit are:


$$\left\{ \begin{array}{c} R_{1}I_{1}+j\omega L_{1}I_{1}-j\omega MI_{2}=U_{1};\\ R_{2}I_{2}+j\omega L_{2}I_{2}-j\omega MI_{1}=0. \end{array}\right.$$ (2-20)

Solving this system of equations is:


$$I_{1}=\frac{U_{1}}{R_{1}+\frac{\omega^{2}M^{2}}{{R_{2}}^{2}+\omega^{2}{L_{2}}^{2}}R_{2}+j\omega \left(L_{1}-\frac{M^{2}\omega^{2}}{{R_{2}}^{2}+\omega^{2}{L_{2}}^{2}}L_{2}\right)}$$ (2-21)

From formula (2–21), the total impedance Z of the coil can be obtained and expressed as:


$$Z=\frac{U_{1}}{I_{1}}=R_{1}+\frac{\omega^{2}M^{2}}{{R_{2}}^{2}+\omega^{2}{L_{2}}^{2}}R_{2}+j\omega \left(L_{1}-\frac{M^{2}\omega^{2}}{{R_{2}}^{2}+\omega^{2}{L_{2}}^{2}}L_{2}\right)$$ (2-22)

R and equivalent inductive reactance XL when the probe coil is placed on the test piece are:


$$\left\{ \begin{array}{c} R=R_{1}+\frac{\omega^{2}M^{2}}{{R_{2}}^{2}+\omega^{2}{L_{2}}^{2}}R_{2};\\ X_{L}=\omega \left(L_{1}-\frac{\omega^{2}M^{2}L_{2}}{{R_{2}}^{2}+\omega^{2}{L_{2}}^{2}}\right). \end{array}\right.$$ (2-23)

From the formula, it can be concluded that when the test piece is a non-ferromagnetic material or a hard magnetic material, the eddy current effect will have a major impact on the equivalent inductance in the coil, thereby reducing the equivalent inductance of the probe. If the test piece is a soft magnetic material, the static magnet electric effect will mainly affect the equivalent inductance in the coil. As the probe approaches the test piece, the equivalent inductance of the probe will increase.

The concept of apparent impedance is introduced here [28], and from formula (2–23) we get:


$$\left\{ \begin{array}{c} R_{X}=R=R_{Z}+R_{1};\\ X_{S}=X_{Z}+X_{1} \end{array}\right.$$ (2-24)

so:


$$\left\{ \begin{array}{c} R_{Z}=\frac{\omega^{2}M^{2}}{{R_{2}}^{2}+\omega^{2}{L_{2}}^{2}}R_{2};\\ X_{Z}=-\frac{\omega^{3}M^{2}L_{2}}{{R_{2}}^{2}+\omega^{2}{L_{2}}^{2}} \end{array}\right.$$ (2-25)

The impedance of the probe coil changes, and this change can be expressed as the reduced impedance (Z_Z_ = R_Z_ + X_Z_). We define the sum of this reduced impedance and the impedance of the primary coil as the apparent impedance (Z_S_ = R_S_ + X_S_). Applying the concept of apparent impedance, we can assume that changes in current or voltage in the primary circuit are due to changes in the circuit’s apparent impedance. This impedance change in the primary circuit can be used to understand the effect of the secondary coil on the primary, and thus infer the impedance change in the secondary circuit.

### 2.3 Skin effect

The related eddy current testing problem, EC is generated by the attenuated magnetic field induction, which will attenuate the EC inside the conductor specimen. This phenomenon is called the skin effect. Under this effect, the current gradually attenuates with increasing depth, and the current is obviously concentrated on the surface of the specimen. The distance defined by the EC penetration into the conductor is called the penetration depth, and the penetration depth defined by the attenuation of the EC density to 1/ *e* (about 36.8%) of its surface value is called the standard penetration depth or skin depth [29]. The calculation formula for the EC penetration depth is:


$$\delta =\sqrt{\frac{\rho }{\pi \mu f}}$$ (2-26)

Where *δ* is called the depth that eddy current can penetrate into the material (m); ρ is called the resistivity of the material (Ω·m); *f* is called the frequency of the alternating current (Hz); *and μ* is called the magnetic permeability of the conductor (H/m).

## 3. Variable parameter eddy current testing model for cylindrical hole defects

### 3.1. Geometric modeling

**Fig 3:**
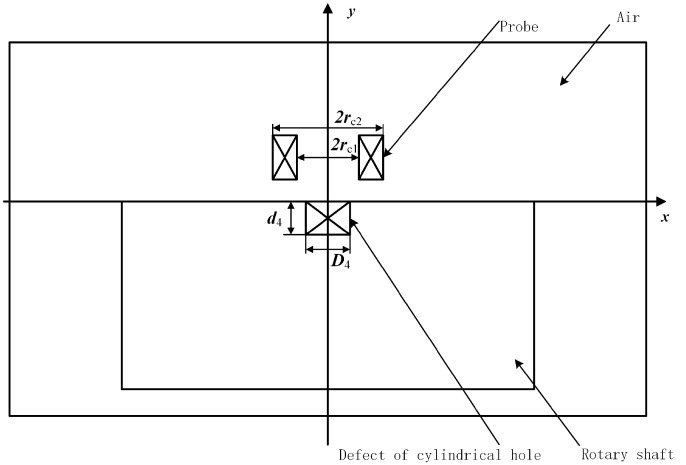
Equivalent model of variable parameter defect detection system for cylindrical hole defects (at the axial section).

In a time – varying magnetic field environment, conductors and other lossy materials generate large amounts of induced currents. This can be modeled using the AC/DC module in COMSOL to establish a corresponding equivalent model. When the corrosion defect is a cylindrical hole, an equivalent model of the variable-parameter defect detection system is established as shown in Fig 3. Here, D4 is the bottom diameter of the cylindrical hole defect, d4 is the defect depth, rc1 is the inner diameter of the coil, set to 2 mm, and rc2 is the outer diameter of the coil, set to 4 mm. To simulate realistic testing conditions, the lift-off distance h between the probe coil and the shaft surface was set to 1.0 mm. The model uses dual boundary constraints: the first type is a forced boundary, corresponding to the outer perimeter of the plane domain in the figure, which is used to limit the solution region; the second type is a natural boundary, which characterizes the interface between different media. If these boundary conditions are set to continuously change over time, the extreme values of the system function will adaptively satisfy all constraints.

### 3.2. Material selection

Air is used as the material for the gas field, copper is used as the material for the coil, and stainless steel is used as the material for the test piece. The relative magnetic permeability *μr*, electrical conductivity *σ*, and relative dielectric constant ε of the selected materials are shown in Table 1.

**Table 1:** Material parameters.

Material	*μr*	*σ*/ (S•m ^−1^)	*ɛ*
Air	1	0	1
Copper	1	5.998 × 10 ^7^	1
Stainless steel	100	1.137 × 10 ^6^	1

### 3.3. Adding physics

COMSOL offers many modules here, we select the “Low-Frequency Electromagnetic Fields” module based on the requirements of the model. The coil type is set to “Uniform Multi-Turn Coil” to easily determine the number of turns and applied current. The number of turns is specified as 500, and the excitation current signal is set to 0.5 A. To simulate an infinitely large space and effectively truncate the computational domain, this paper applies a “magnetic insulation” boundary condition to the outermost boundary of the air domain, forcing the magnetic field lines to be parallel to the boundary. Simultaneously, a Dirichlet boundary condition is applied to the coil model, specifying the magnetic vector potential as zero, to ensure the convergence and uniqueness of the electromagnetic field solution.

To clearly illustrate the specific settings of the simulation model, this paper summarizes the key geometric dimensions and electromagnetic excitation parameters, as shown in Table 2.

**Table 2:** Key parameter settings for eddy current testing simulation models.

Parameter	Symbol	Value	Unit
Excitation Frequency	f	1	kHz
Current Amplitude	I	0.5	A
Number of Turns	N	500	–
Coil Inner Radius	rc1	2.0	mm
Coil Outer Radius	rc2	4.0	mm
Lift-off Distance	h	1.0	mm
Shaft Radius	R	50.0	mm

### 3.4. Mesh generation

**Fig 4:**
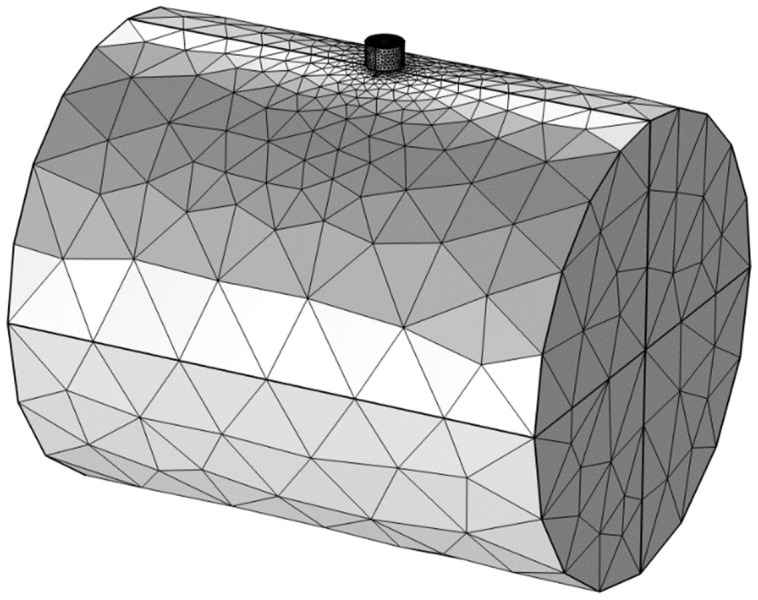
Schematic diagram of mesh partitioning.

Meshing is a crucial step in software simulation solutions. The quality of meshing is crucial to the accuracy and correctness of the results. Finite element analysis is the foundation of simulation, and the research object is the direct purpose of finite element meshing. The most commonly used mesh in eddy current model simulations is the free tetrahedron mesh. Because eddy current probes are relatively precise, extremely fine meshing is required to generate a denser tetrahedron mesh, ensuring more accurate calculation results.The specific parameters are set as follows: maximum element size is 4 mm, minimum element size is 0.001 mm, maximum element growth rate is controlled at 1.3, curvature factor is set to 0.2, and narrow region resolution is 1. Conventional meshing is often used for rotating shafts due to their relatively large size. The specific parameters are set as follows: maximum element size is 20 mm, minimum element size is 0.001 mm, maximum element growth rate is 1.5, curvature factor is 0.6, and narrow region resolution is 0.5. The meshing model is shown in Fig 4.

### 3.5. Solving and post-processing

**Fig 5:**
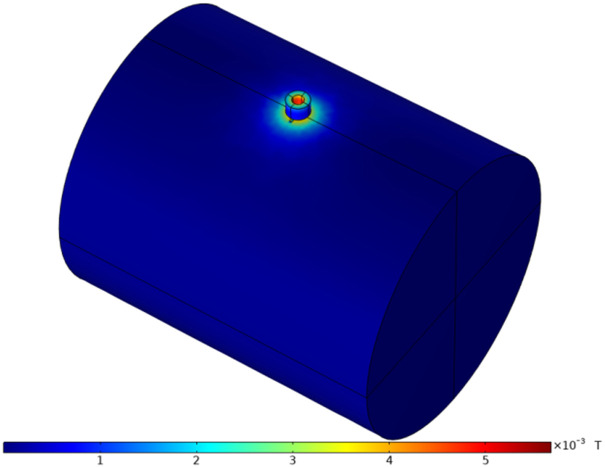
Magnetic induction intensity energy distribution diagram on the shaft surface.

Enter the excitation frequency in the model and set the correct solution. After meshing, solve for the variable defect parameters in the model to obtain the corresponding magnetic field distribution. Then, analyze the relationship between the magnetic field and the defect geometry (bottom diameter, depth). This allows you to determine the electromagnetic properties near the defect. You can also analyze the distribution of magnetic induction intensity in the shaft under different frequencies to study its impact on the electromagnetic properties near the defect. A spatial view of the magnetic induction intensity distribution is shown in Fig 5.

**Fig 6:**
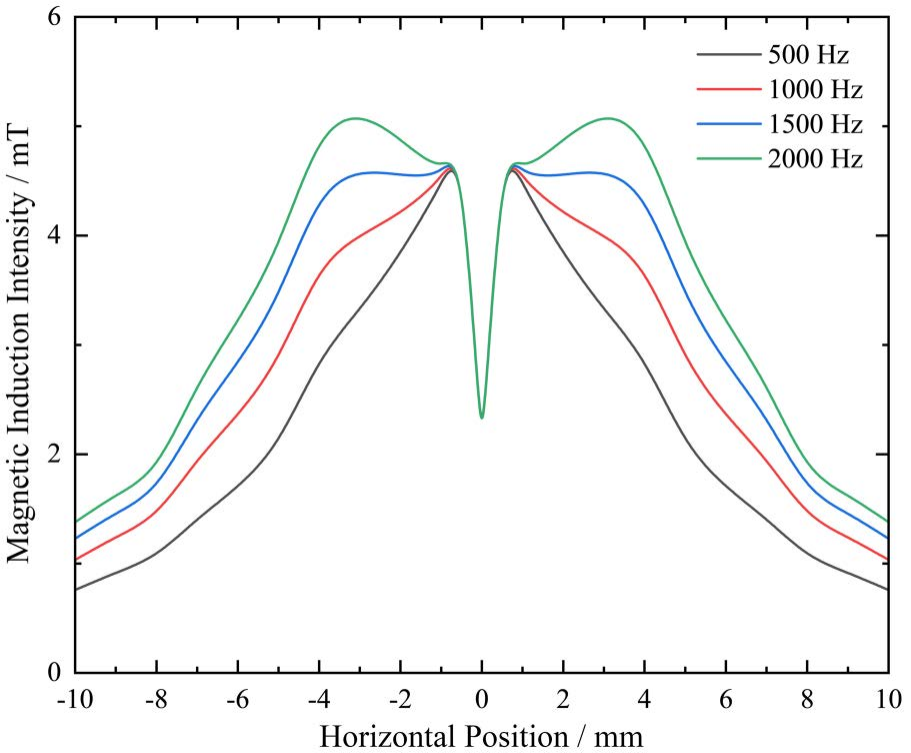
Magnetic induction intensity distribution curve of the horizontal shaft surface.

The magnetic cloud image near the defect is generated by solving the problem. Taking a simple defect with a bottom diameter and depth of 1 mm as an example, after the solution is completed, the change in magnetic induction intensity in the horizontal direction is first explored. The change in magnetic induction intensity in the rotating shaft at this time can be observed in the top view direction, as shown in Fig 6. The trend of magnetic induction intensity change is similar at different frequencies. It can be -seen from the figure that the maximum magnetic induction intensity is close to the center of the probe coil, while the minimum magnetic induction intensity is located farther away from the probe, that is, at the edge.

**Fig 7:**
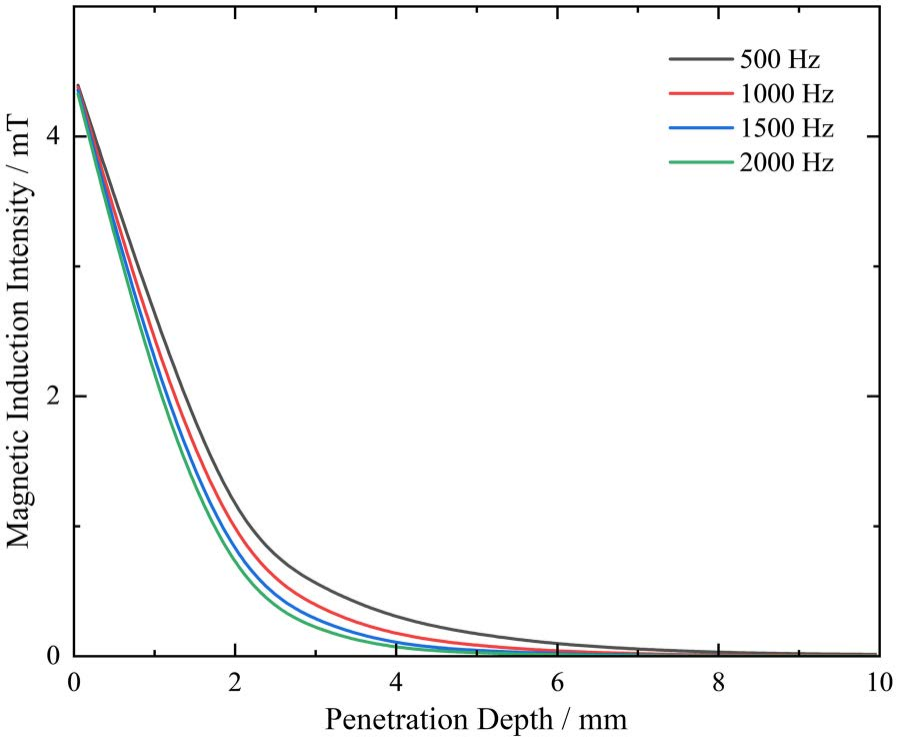
Curve diagram of magnetic induction intensity distribution on the vertical shaft surface.

Select a three-dimensional cross-section perpendicular to the horizontal plane and observe the change in magnetic induction intensity in the vertical direction. A curve is obtained, as shown in Fig 7. This variation pattern can be used to analyze the relationship between magnetic induction intensity and penetration depth. The trend of magnetic induction intensity change is similar at different frequencies. The graph shows that the maximum magnetic induction intensity is at the surface of the shaft and decreases with increasing depth. This phenomenon is often caused by the skin effect. Since a frequency that is too small can affect the sensitivity of the probe, the subsequent research will set the frequency at 1 kHz.

### 3.6. Results and Discussion The magnetic field distribution law of cylindrical holes with different bottom diameters

The depth of the cylindrical hole defect d4 = 0.5 mm is kept unchanged, and the value of the bottom diameter D4 of the cylindrical hole defect is changed to study its influence on the MF of the surface around the defect. The factors affecting the MF distribution law of different bottom diameters are mainly reflected in the amplitudes of the horizontal and vertical magnetic induction intensities and their respective phases. The range of variation of the bottom diameter is D4 = 0.01 mm (assuming a defect-free state), D4 = 1 mm, D4 = 2 mm, D4 = 4 mm, D4 = 6 mm, and D4 = 12 mm.

**Fig 8:**
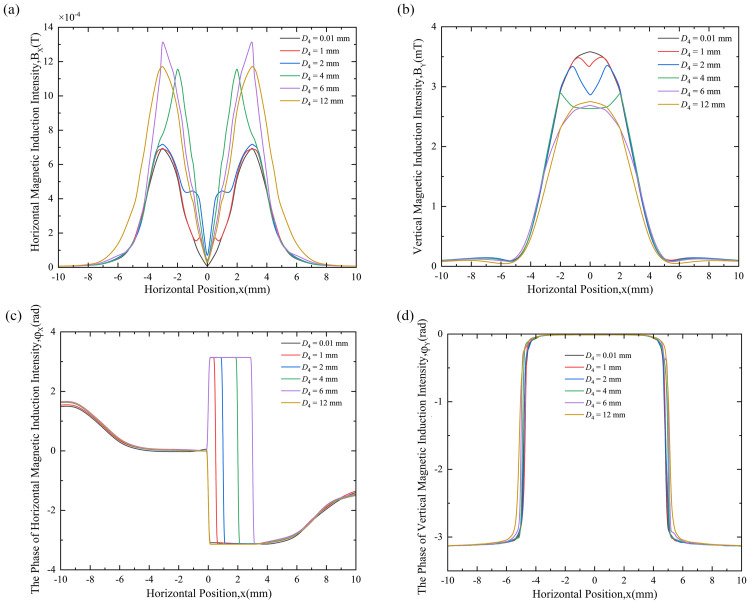
Magnetic field variation curves for 6 cylindrical hole defects with different bottom diameters: (a) HMII variation curve, (b) VMII variation curve, (c) PHMII variation curve, (d) PV MII variation curve.

Distribution patterns of HMII, VMII, PHMII and PVMII under different bottom diameters of cylindrical hole defects are shown in Fig 8. As shown in (a), (b), (c), and (d).

Fig 8(a) shows the variation curve of HMII under different bottom diameters.From the variation of MF under different bottom diameters in the figure, the following rules can be drawn: (1) Under different bottom diameters, the distribution of HMII is obviously different. Compared with the case where the bottom diameter is 0.01 mm, the HMII distribution under other bottom diameter values has huge differences. Either there is a symmetrical peak or the original peak becomes larger. (2) When D4 = 0.01 mm, HMII gradually increases as the distance from the zero coordinate increases. It slowly decreases until it reaches the maximum value of 3 mm (equivalent radius) and finally approaches 0. (3) When D4 = 1 mm and 2 mm, two pairs of peaks appear in the HMII change curve. The spacing between the pair of peaks close to the zero coordinate is equal to the corresponding bottom diameter, which increases with the increase of the bottom diameter value. The spacing between the pair of peaks farther away is equal to 6 mm (twice the equivalent radius), and this pair of peaks does not change with the bottom diameter and remains basically unchanged. (4) When D4 = 4 mm, 6 mm and 12 mm, the HMII curve has only one pair of peaks symmetrical with the zero coordinate, and each peak appears 3 mm away from the zero coordinate, which is similar to the defect-free change curve, but at this time the peaks of these bottom diameters will be higher than the peaks when D4 = 0.01 mm.

As shown in Fig 8(a),the characteristic peaks are defined as a pair of peaks close to the zero coordinate, distinguishing them from the original peaks located at ±3 mm. The characteristic peak distance is referred to as the distance between the two characteristic peaks. This allows for quantitative analysis of the bottom diameter and quantitatively characterizes the size of the bottom diameter. By comparing the HMII curve with the defect-free state, the presence of a defect can be qualitatively determined. Moreover, the presence or absence of the characteristic peak further reveals the relationship between the bottom diameter and 6 mm: if the bottom diameter is less than 6 mm, the characteristic peak appears in the HMII curve; if the bottom diameter is greater than 6 mm, the characteristic peak disappears, but the original peak value increases significantly, thus still allowing the defect to be identified.

Fig 8(b) shows the variation curve of VMII under different bottom diameters. From the variation of MF under different bottom diameters in the figure, the following rules can be drawn: (1) The distribution of VMII under different bottom diameters is obviously different. Compared with the case of 0.01 mm bottom diameter, the distribution of VMII under other bottom diameter values is very different, and a dip occurs at the zero coordinate. (2) When D4 = 0.01 mm, the peak is at the zero coordinate, and V MII gradually shows a decreasing trend as the distance from the zero coordinate increases. There will be a phenomenon of slight increase and then decrease later. (3) When D4 *=* 1 mm, 2 mm and 4 mm, the VMII change curve has a valley value at the zero coordinate. Its value decreases with the increase of the bottom diameter value. (4) When D4 = 6 mm and 12 mm, the VMII curve changes similarly to the defect-free change curve, and the peak value appears at the zero coordinate respectively, but the peak value of these two bottom diameters will be smaller than the peak value when D4 = 0.01 mm.

As shown in Fig 8(b), the presence of a defect can be qualitatively determined by comparing the VMII curve with that of a defect-free state: if there is a concave at the zero coordinate, or if there is no concave but the peak value drops significantly, then the defect is present; otherwise, the defect is absent. Furthermore, the defect size can be inferred from the curve’s variation: when the bottom diameter is < 6 mm, the VMII curve is concave at the zero coordinate; when the bottom diameter is ≥ 6 mm, there is no concave at the zero coordinate but the peak value drops significantly.

Fig 8(c) shows the change curve of PHMII under different bottom diameters. From the change of MF under different bottom diameters in the figure, the following rules can be drawn: (1) The distribution of PHMII is very different under different bottom diameters. When *D*4 = 1 mm, 2 mm, 4 mm and 6 mm, compared with the case where the bottom diameter is 0.01 mm, the distribution of PHMII curve is very different, and the phase remains at π in a certain period of time. And as the bottom diameter continues to increase, the hysteresis phenomenon becomes more obvious, and the phase interval of π widens. (2) When *D*4 = 12 mm, the phase of PHMII no longer lags, and there is no time period where the phase remains at π. At the same time, the change curve of PHMII is basically consistent with the change curve under the defect-free situation.

Fig 8(c) shows that PHMII information can be used to detect cylindrical hole defects. When the defect is not large, the presence of the cylindrical hole defect can be qualitatively determined by comparing it with the HMII phase change curve in a defect-free state. When the defect is small, if there is no phase lag, there is no defect; otherwise, there is a defect. The wider the phase retention interval π during the lag period, the larger the bottom diameter; twice the length of the lag period interval is the bottom diameter, allowing for quantitative assessment. Regarding the hysteresis phenomenon, the phase lag is caused by the presence of surface defects. Due to the eddy current effect, the continuously distributed steady-state induced magnetic field that should have formed near the surface is affected by the defects. This is further confirmed by the quantitative relationship between the hysteresis interval and the defect size; the phase lag interval is located between the center of the defect and its boundary. When the defect is large, the cylindrical hole defect exceeds the coil’s primary sensitive area, and the phase information is invalid, making it impossible to infer the presence of the defect.

Fig 8(d) shows the variation curves of PVMII for different base diameters. From the variation of MF for different base diameters in the figure, it can be concluded that the PVMII variation curves for different base diameters are very small and difficult to distinguish. This shows that PVMII is not sensitive to base diameter and therefore cannot be used as a feature quantity for base diameter detection.

### 3.7. Results and Discussion The magnetic field distribution law at different depths of cylindrical holes

Keeping the bottom diameter of the cylindrical hole defect D4 = 2 mm unchanged, the influence of the depth d4 of the cylindrical hole defect on the surface MF around the defect is studied. The factors affecting the MF distribution law at different depths are mainly reflected in the amplitudes of the horizontal and vertical magnetic induction intensities and their respective phases. The depth variation range is d4 = 0.01 mm (assuming a defect-free state), d4 = 0.5 mm, d4 = 1 mm, d4 *=* 2 mm, d4 = *3* mm, and d4 *=* 6 mm.

**Fig 9:**
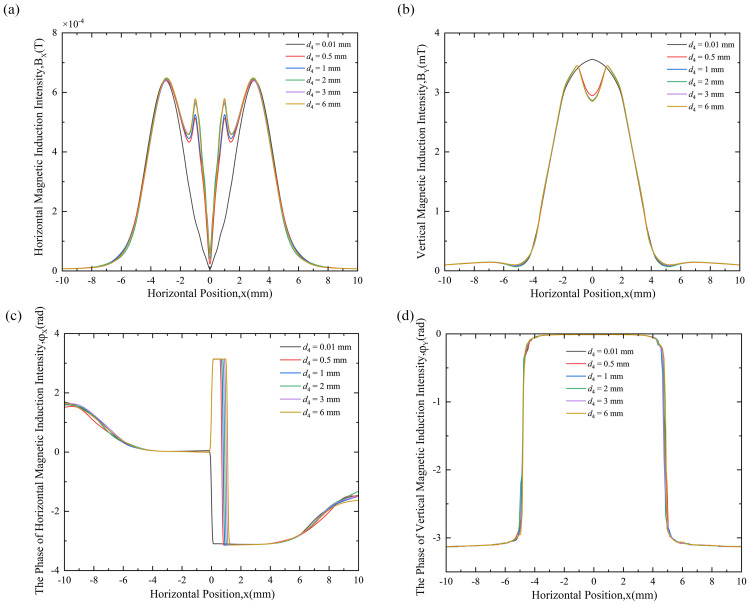
Magnetic field variation curves at different depths of cylindrical hole defects: **(a)** HMII variation curve, **(b)** VMII variation curve, **(c)** PHMII variation curve, **(d)** PVMII variation curve.

The distribution patterns of HMII, VMII, PHMII, and PVMII at different depths of cylindrical hole defects are shown in Fig 9(a), (b), (c), and (d), respectively.

Fig 9(a) shows the variation curve of HMII at different depths. The following rules can be drawn from the variation of MF at different depths in the figure: (1) The distribution of HMII varies significantly according to different depth values. Compared with the case of a depth of 0.01 mm, the HMII distribution at other depth values d4 = 0.5 mm, 1 mm, 2 mm, 3 mm and 6 mm has huge differences. The variation curves are obviously different. There is a pair of symmetrical peaks. The peak close to the zero coordinate is still defined as the characteristic peak (2). Different from the defect-free state, the characteristic peak of each depth will increase with the depth, and then tend to be stable after the depth increases to a certain extent. [3] When the defect depth is different, the distance between the pair of characteristic peaks close to the zero coordinate remains unchanged, maintaining at 2 mm, and the distance between the pair of peaks farther away is also basically the same, stable at 6 mm, and the variation curves are not much different.

Fig 9(a) shows that HMII can be used as a characteristic variable to detect the depth of cylindrical hole defects. By comparing the HMII curve with that of a defect-free state, the presence of a defect can be determined. If a characteristic peak appears in the curve, a defect is present; otherwise, no defect exists. The peak size can qualitatively distinguish depth: smaller peaks correspond to smaller depths, while increasing depth leads to larger peaks. When the depth exceeds a certain threshold, the peak saturates and no longer varies with depth, making it impossible to compare the relative depth using the peak size of the characteristic peak. The distance between the characteristic peaks and their positions remain unchanged because the bottom diameter of the cylindrical hole is set to 2 mm, and the characteristic peak distance is equal to the bottom diameter.

Fig 9(b) shows the variation curves of VMII at different depths. From the variation of MF at different depths in the figure, the following rules can be drawn: (1) The distribution of VMII is significantly different according to different depth values. Compared with the case of a depth of 0.01 mm, the distribution of VMII at other depth values d4 *=* 0.5 mm, 1 mm, 2 mm, 3 mm and 6 mm is very different, and the variation curves are obviously different, which is manifested in the concave phenomenon at the zero coordinate of the variation curve. (2) When d4 = 0.5 mm, 1 mm, 2 mm, 3 mm and 6 mm, the concave becomes deeper as the depth increases, and after reaching a certain depth, such as 1 mm, the concave will deepen slightly and gradually tend to a stable value. (3) For the defect-free state, the peak is at the zero coordinate, V The MII gradually decreases as the distance from the zero coordinate increases, and then increases slightly and then decreases.

As shown in Fig 9(b),comparing the VMII curve with the defect -free state allows for a qualitative determination of the presence of a cylindrical hole defect. Specifically, if the curve shows a dip at the zero coordinate, a defect is present; otherwise, the defect is absent. The dip amplitude can be used to distinguish depth, but this only applies to relatively small depths; the greater the depth, the deeper the dip. When the depth is large, exceeding a certain value, a qualitative comparison of the relative depths of the defect cannot be made, and the dips at the zero coordinate are essentially the same, making them indistinguishable.

Fig 9(c) shows the PHMII curves at different depths. The MF variations at different depths in this figure reveal the following pattern: the PHMII distribution varies significantly at different depths. When D4 = 1 mm, 2 mm, 4 mm, and 6 mm, the PHMII curve distribution differs significantly compared to the case with a base diameter of 0.01 mm. For a certain period of time, the phase remains at π. Furthermore, as the base diameter increases, the hysteresis increases slightly, and the phase interval of π increases.

Fig 9(c) shows that PHMII variation can be used to detect cylindrical hole defects. The physical mechanism is consistent with the aforementioned analysis of the base diameter, and the phase lag originates from the perturbation of the surface-induced magnetic field by the defect. By comparing the PHMII variation curve with the defect-free state, the presence of a crack defect can be qualitatively determined. If phase lag occurs, a defect is present; otherwise, no defect is present. Similar to cylindrical hole defects with different base diameters, the relative depth can be qualitatively compared by comparing the size of the interval where the phase remains at π. However, this is only a weakened qualitative comparison; this interval length cannot be used to quantitatively infer the depth, unlike the case of cylindrical hole defects with different base diameters.

As shown in Fig 9(d), the PVMII curves for cylindrical hole defects at different depths are very different and difficult to distinguish. Similar to the bottom diameter, PVMII is also insensitive to defect depth, so PVMII cannot be used as a feature quantity for depth detection.

### 3.8. Experimental verification

**Fig 10:**
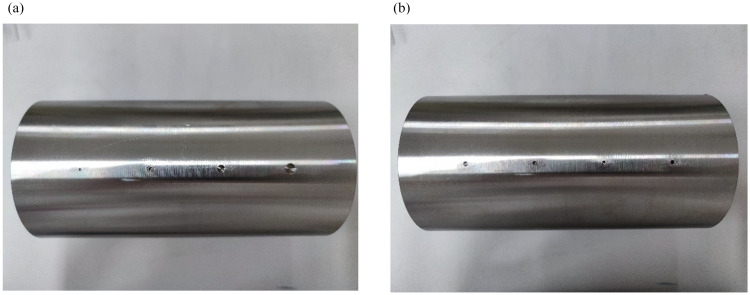
The shaft to be tested has different surface defects: (a) Cylindrical holes of different diameters; (b) Cylindrical holes of different depths.

This section examines specimens with cylindrical hole defects and cracks of varying bottom diameters or depths. Fig 10 show images of the machined shafts with different surface defects. The shaft surface defects are of two types: cylindrical hole defects with bottom diameters of 1 mm, 2 mm, 4 mm, and 6 mm; and cylindrical hole defects with depths of 1 mm, 2 mm, 3 mm, and 6 mm. Experimental verification is conducted. The coil voltage, which is easily and accurately obtained, is selected as the observed quantity. If the measured voltage signal is consistent with the simulation result, the simulation model is reliable. Since the voltage and magnetic field quantities in the simulation are different outputs obtained under the same solution environment, experimental validation of the model indirectly verifies the accuracy of the magnetic field analysis.

**Fig 11:**
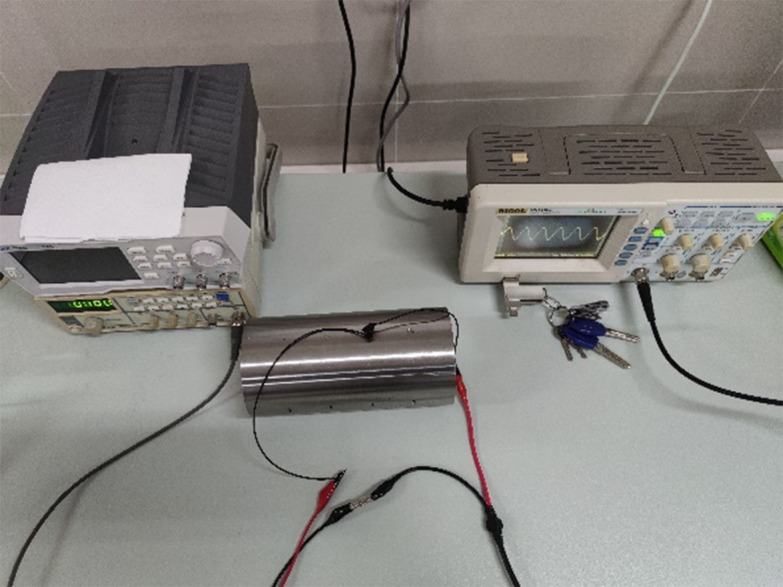
Eddy current testing experimental platform.

The experimental platform mainly consists of a detection device and a test piece, as shown in Fig 11. The detection device mainly includes an excitation source, a coil, an oscilloscope, etc.

During the experiment, all parameters were consistent with the simulation. The coil moving on the shaft surface passed through the defect location. The zero coordinate was the center of the defect. The voltage signal change within the coil was plotted and compared to the simulation data.

**Fig 12:**
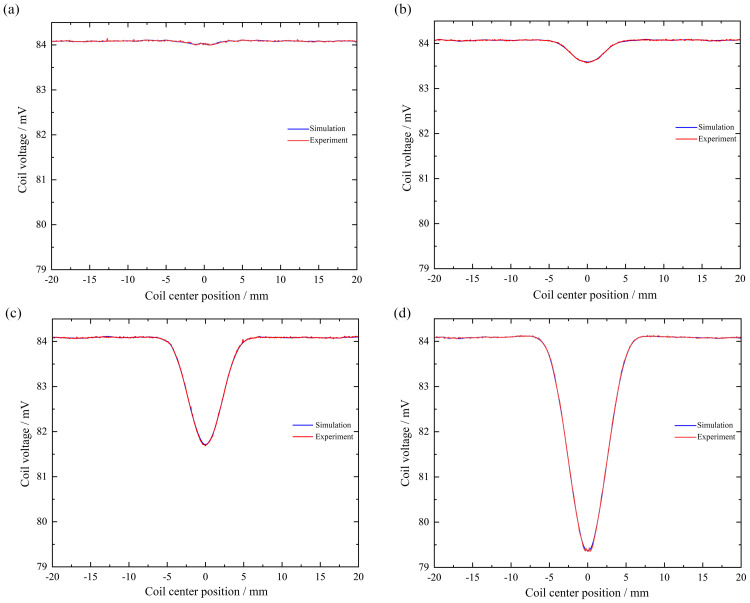
Simulation and experimental comparison results of coil voltage signals under different cylindrical hole bottom diameters: (a) Bottom diameter is 1 mm; (b) Bottom diameter is 2 mm; (c) Bottom diameter is 4 mm; (d) Bottom diameter is 6 mm.

When the surface defects of the rotating shaft are cylindrical holes with bottom diameters of 1 mm, 2 mm, 4 mm, and 6 mm, the changes in voltage signals in simulation and experiment are shown in Fig 12.

As shown in Fig 12(a), when the diameter of the bottom surface of the cylindrical hole is 1 mm, the voltage signal change curves in the simulation and experiment are almost identical,and a concave portion appears at the defect. The simulated trough value here is 84.02 mV, while the trough value at the concave portion in the experiment is approximately 84.00 mV, the relative error between the two is only 0.02%, indicating a high degree of agreement between the values and validating the accuracy of the model under these operating conditions.

As shown in Fig 12(b), when the diameter of the bottom surface of the cylindrical hole is 2 mm, the voltage signal change curves in the simulation and experiment are basically consistent, and a concave area appears at the defect. The simulated trough value here is 83.58 mV, and the trough value at the concave area in the experiment is about 83.60 mV, the relative error is approximately 0.02%, further demonstrating the model’s prediction accuracy across different pore sizes.

As shown in Fig 12(c), when the bottom diameter of the cylindrical hole is 4 mm, the voltage signal change curves in the simulation and experiment are basically consistent, and a concave area appears at the defect. The simulated trough value here is 81.69 mV, and the trough value at the concave area in the experiment is about 81.70 mV, the difference between the two is extremely small (error < 0.02%), and the experimental results strongly support the simulation conclusions.

As shown in Fig 12(d), when the bottom diameter of the cylindrical hole is 6 mm, the voltage signal change curves in the simulation and experiment are basically consistent, and a concave portion appears at the defect. The simulated trough value here is 79.35 mV, and the trough value at the concave portion in the experiment is approximately 79.30 mV, the relative error is approximately 0.05%, which is still within a very low range, indicating that the model remains applicable to large-aperture defects.

**Fig 13:**
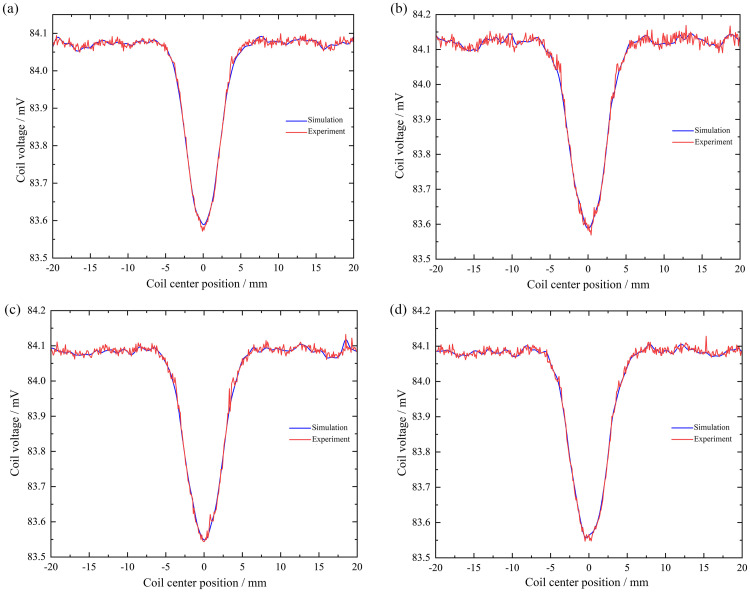
Simulation and experimental comparison results of coil voltage signals at different cylindrical hole depths: (a) Depth is 1 mm; (b) Depth is 2 mm; (c) Depth is 3 mm; (d) Depth is 6 mm.

1 mm, 2 mm, 3 mm, and 6 mm, respectively, the changes in voltage signals in simulation and experiment are shown in Fig 13.

As shown in Fig 13(a), when the depth of the cylindrical hole is 1 mm, the voltage signal change curves in the simulation and experiment are basically consistent,and a concave phenomenon will appear at the defect. Here, the trough value in the simulation is 83.58 mV, and the trough value in the experiment is about 83.56 mV, the relative error was only 0.02%, which validated the model’s ability to analyze shallow defects.

As shown in Fig 13(b), when the depth of the cylindrical hole is 2 mm, the voltage signal change curves in the simulation and experiment are basically consistent, and both show a concave phenomenon at the defect. Here, the trough value in the simulation is 83.58 mV, and the trough value at the concave part in the experiment is about 83.57 mV, the error is approximately 0.01%, indicating a very high degree of agreement between the values.

As shown in Fig 13(c), when the depth of the cylindrical hole is 3 mm, the voltage signal change curves in the simulation and experiment are basically consistent, and both will have a concave phenomenon at the defect. Here, the trough value in the simulation is 83.54 mV, and the trough value in the experiment is about 83.53 mV, the relative error is less than 0.02%, which strongly supports the simulation results.

As shown in Fig 13(d), when the depth of the cylindrical hole is 6 mm, the voltage signal change curves in the simulation and experiment are basically consistent, and both will show a concave phenomenon at the defect. Here, the trough value in the simulation is 83.57 mV, and the trough value in the experiment is about 83.56 mV, the error is approximately 0.01%, and the values are highly consistent, which verifies the accuracy of the model’s detection.

Although the simulation and experimental results show excellent numerical agreement (maximum relative error not exceeding 0.05%), small discrepancies still exist. These are mainly attributed to unavoidable systematic errors during the experiment, such as slight fluctuations in the probe lift-off distance, dimensional tolerances due to manual processing defects, and non-uniformity of the material’s electromagnetic parameters. However, given that the errors are all within a very low range and the trends are in complete agreement, these results fully demonstrate the accuracy and reliability of the simulation model proposed in this paper.

In summary, the voltage changes in simulation and experiment under different types of defects are basically consistent, which verifies the accuracy of the model and indirectly verifies the correctness of the magnetic field correlation analysis.

## 4. In conclusion

This paper simulates the distribution of HMII, VMII, PHMII, and PVMII near a shaft surface defect based on defect parameters (bottom diameter and depth) for a cylindrical hole with corrosion geometry. Analysis of these distribution patterns yields the following results: By observing the variations in magnetic field components, we explored the influence of defect geometry and shape on the spatial magnetic field response. The accuracy of the magnetic field analysis is indirectly verified through voltage-signal variation experiments. Cylindrical hole defects (corrosion defects) were also studied. The results showed that, firstly, HMII and its phase (PHMII) can be used for quantitative evaluation, exhibiting significant geometric feature correlations. Quantitative assessment of the defect bottom diameter can be achieved by analyzing the characteristic peak spacing of HMII or the phase lag width of PHMII. Secondly, HMII and VMII can be used for qualitative evaluation of depth; the amplitude changes of these two parameters can effectively determine the presence of cylindrical hole defects and qualitatively assess the relative size of the defect depth. However, it should be noted that the signal tends to saturate when the depth exceeds a certain threshold, which limits its ability to distinguish between defects at greater depths. Finally, PVMII is insensitive to both bottom diameter and defect depth, making it incapable of quantitatively or qualitatively assessing these characteristics. It can serve as a “negative control” benchmark for the detection system, highlighting the high sensitivity of the horizontal component (HMII) to defect edge effects. The effect of HMII, VMII, and their respective phases in assessing the size of the defect is verified.

## References

[1] Zhang H, Zhang Z, Chen Y. Application progress and prospects of non-destructive detection technology for industrial casting defects. Acta Automatica Sinica. 2022;48(04):935–56.

[2] Huang S, Peng L, Sun H, et al. Review of nondestructive testing and online monitoring technology for aircraft engine blade defects. Measurement and Control Technology. 2023;42(05):1–11.

[3] Li Y, Wang K, Liu C. Current status of surface defect detection of metal sheets. Materials Review: Nanomaterials and New Materials. 2011;25(2):4.

[4] Xu T. Research on bridge eddy current detection system based on impedance analysis method. North University of China. 2009.

[5] Machado MA, Rosado LFSG, Mendes NAM, Miranda RMM, dos Santos TJG. New directions for inline inspection of automobile laser welds using non-destructive testing. Int J Adv Manuf Technol. 2021;118(3–4):1183–95. doi: 10.1007/s00170-021-08007-0

[6] Sergieva-Chollet N, Decitre JM, Fermon C. Development of eddy current probes based on magnetoresistive sensors arrays. In: Proceedings of the AIP Conference, 2014.

[7] Ye C, Wang Y, Wang M. Frequency domain analysis of magnetic field images obtained using TMR array sensors for subsurface defect detection and quantification. NDT and E International. 2020;116.

[8] Rajamäki J, Vippola M, Nurmikolu A, Viitala T. Limitations of eddy current inspection in railway rail evaluation. Proceedings of the Institution of Mechanical Engineers, Part F: Journal of Rail and Rapid Transit. 2016;232(1):121–9. doi: 10.1177/0954409716657848

[9] Cao B, Li X, Wang M, Fan M. Analytical modelling and simulations for high-frequency eddy current testing with planar spiral coils. Nondestructive Testing and Evaluation. 2020;36(2):195–208. doi: 10.1080/10589759.2020.1714614

[10] Mizukami K. Evaluation of size of surface and subsurface waviness in carbon fiber composites using eddy current testing: a numerical study. Advanced Composite Materials. 2017;27(6):589–604. doi: 10.1080/09243046.2017.1417092

[11] Simin W. Array eddy current nondestructive testing system based on spin sensing and magnetic shielding structure. Hangzhou Dianzi University, 2022.

[12] Xiong L, Liu X, Zhang Y, et al. Identification of high-speed railway rail scratches based on eddy current detection. Railway Construction. 2022;62(05):8–12.

[13] Hongfu C. Research on eddy current nondestructive testing technology of bearing roller based on PCB planar spiral coil. Xiamen University. 2022.

[14] Xiao F, Huang Y, Zhang B, et al. Experimental study on eddy current detection of lead seal defects in high-voltage cable accessories. China Metal Bulletin. 2022;(07):216–8.

[15] Li L. Design and implementation of electromagnetic field simulation platform for RCF electromagnetic eddy current detection of high-speed railway tracks. University of Electronic Science and Technology of China. 2021.

[16] Zeng H, Ye B, Zhang Y. Finite element simulation study on vertical eddy current detection of delamination defects in unidirectional carbon fiber composite materials. Journal of Sensor Technology. 2020;33(01):45–50.

[17] Ren S, Li Y, Zhang X, et al. Visualization method of subsurface corrosion defects of aviation structures based on magnetic field gradient pulsed eddy current testing. Journal of Air Force Engineering University (Natural Science Edition). 2019;20(03):84–9.

[18] Wang X, Fu Y. Pulsed eddy current detection of corrosion defects in ferromagnetic flat components. Failure Analysis and Prevention. 2018;13(06):358–61.

[19] Jiang F. Study on eddy current magnetic field analytical model and defect electromagnetic characteristics. Shanghai University. 2019.

[20] Wang L, Cai W, Chen Z. Quantitative evaluation of stress corrosion cracking based on crack conductivity model and intelligent algorithm from eddy current testing signals. Nondestructive Testing and Evaluation. 2019;35(4):378–94. doi: 10.1080/10589759.2019.1699085

[21] Li Z, Tian W. Simulation study on eddy current detection of corrosion defects on inner wall of pipeline. Computer Simulation. 2018;35(07):331–4.

[22] Versaci M, Cacciola M, Laganà F, Angiulli G. Analysis of Acoustic Wave Propagation in Defective Concrete: Evolutionary Modeling, Energetic Coercivity, and Defect Classification. Applied Sciences. 2025;15(21):11378. doi: 10.3390/app152111378

[23] Shuguang T. Application of eddy current detection technology in subway structure flaw detection. Xiamen University of Technology, 2022.

[24] Changyu S, Xiaochu Z, Xiang Z. Research on simulation and test of shaft voltage of traction motor of EMU. Railway Locomotive and EMU. 2021;2021(09):20–3.

[25] Shan W, Li D, Liu S, Song M, Xiao S, Zhang H. An artificial immune classification method with deep feature enhancement and dynamic memory cells optimization. Expert Systems with Applications. 2026;304:130850. doi: 10.1016/j.eswa.2025.130850

[26] Qiu J, Qi Y, Xu X. Simulation of eddy current probe coil based on COMSOL finite element method. Journal of Lanzhou Jiaotong University. 2021.

[27] Chen F. Modeling and simulation analysis of defect eddy current testing for titanium alloy sheet. Kun University of Science and Technology. 2016.

[28] Zheng J. Research on the application of eddy current testing technology in multilayer thickness testing. Zhejiang University. 2003.

[29] Yang J, Jiao S, Zeng Z, Lin J, Zhao J. Skin effect in eddy current testing with bobbin coil and encircling coil. PIER M. 2018;65:137–50. doi: 10.2528/pierm18011904

